# Assessment of physical activity and its facilitators and barriers among Syrian refugees living in Amman City, Jordan: a cross-sectional study

**DOI:** 10.1186/s12889-022-14064-1

**Published:** 2022-09-12

**Authors:** Yasue Yoshino, Miho Sato, Ibraheem Abu-Siam, Nadine Khost, Sumihisa Honda, Ahmad T. Qarawi, Osama Gamal Hassan, Nguyen Tien Huy, Yasuhiko Kamiya

**Affiliations:** 1 School of Tropical Medicine and Global Health, Nagasaki University, Nagasaki, Japan; 2 United Nations High Commissioner for Refugees, Amman, Jordan; 3 Blumont, Amman, Jordan; 4 Graduate School of Biomedical Sciences, Nagasaki University, Nagasaki, Japan; 5 Fleetwood Speciality Pharmacy, New York, USA; 6 Online Research Club, Nagasaki, Japan; 7 Cardiology Department, El Zaitoun Specialized Hospital, Cairo, Egypt

**Keywords:** Syrian Refugees, Urban refugees, Jordan, Health, Physical activity, Physical inactivity, IPAQ, Non-communicable diseases, Facilitators, Barriers

## Abstract

**Background:**

Physical inactivity is one of the major risk factors for non-communicable diseases. Few studies about physical activity have been conducted among refugees from neighbouring countries. Given changes in the situation of Syrians, assessment of physical activity among Syrian refugees is required to understand their situation. This study aimed to evaluate the degree of self-reported physical activity and to identify perceived facilitators of and barriers to physical activity among Syrian refugees living in Amman, Jordan, in 2017.

**Methods:**

This community-based cross-sectional study was conducted using a structured questionnaire and the short form of the International Physical Activity Questionnaire. Participants were eligible for the study if they were Syrian refugees aged 18–64 years, living in Amman city, and were either registered with the United Nations High Commissioner for Refugees, waiting for their registration, or had a service card issued by the Jordanian Ministry of Interior. The relationship between physical activity level and sex was assessed using the chi-square test and Cochran–Armitage tests. The Mann–Whitney U test was performed to assess the relationship between the median metabolic equivalent scores of physical activity and gender. Backward stepwise logistic regression analysis was used to analyse the association between predictors of physical inactivity and physical activity level.

**Results:**

Among the 173 participants, the majority (91.9%) reported moderate to a high level of physical activity, and 8.1% were physically inactive. The metabolic equivalent scores for the walking activity of males (median: 1039.5, IQR: 0, 2772) was significantly higher than that of females (median: 396, IQR: 0, 1188) (*p* < 0.01). “Perceived change in the amount of physical activity” was a significant predictor of physical inactivity (adjusted OR = 3.00; 95%CI: 1.27–7.26). Common facilitators of physical activity were “psychological wellbeing”(49.7%) and “prevent diseases”(46.8%). The greatest barriers to physical activity were “time limitation”(43.4%) and “high cost”(57.8%).

**Conclusion:**

This study revealed the physical activity level among Syrian refugees in Amman. The perceived facilitators and barriers to physical activity identified among Syrian refugees were similar to those in previous studies conducted among non-refugees. These results provide a valuable baseline for future examinations of physical activity level and to verify its possible facilitators and barriers.

**Supplementary Information:**

The online version contains supplementary material available at 10.1186/s12889-022-14064-1.

## Background

The growing influx of vulnerable populations poses many challenges to host countries [1]. Jordan, which has hosted refugees from various countries and areas for a long time, is no exception. Since 2011, millions of people have fled from Syria to Jordan and other neighbouring countries, and the influx of refugees has overstretched Jordan’s health system [2].

Non-communicable diseases (NCDs) kill 41 million people worldwide each year, and 77% of all NCD deaths occur in low- and middle-income countries [3]. The burden of NCDs such as cardiovascular disease, cancer, chronic lung diseases, and diabetes in the Arab world has increased, with variations between countries of different income levels [4]. Jordan is categorized as a lower-middle-income country, whereas Syria is categorized as a low-income country [5, 6]. Prior to the Syrian conflict that dates back to 2011, NCDs accounted for 77% of the total mortality in Syria in 2008 [7], whereas the mortality rate in Jordan due to NCDs was 76% in 2014 [8]. In addition, as the World Health Organization (WHO) explains that people of all regions and countries are affected by NCDs [3], the risks of developing NCDs among both Syrians and refugees are similar as well. Therefore, Syrian refugees in Jordan are likely to face a continuous high risk of death caused by NCDs.

The results of a survey conducted in 2014 reported that more than half of Syrian refugee households had a member with an NCD [9] and that the impending burden could incur substantial medical costs to the Government of Jordan. Until November 2014, primary and secondary care for Syrian refugees was free at public facilities in Jordan [10]. Since then, government policy has required refugees registered with the Ministry of Interior to pay a subsidized rate for care (similar to that for uninsured Jordanians) [11]. A previous study predicted that NCD diagnoses in Syrian refugees would become an increasing burden on health services in Jordan [9]; therefore, the health system is likely to be overwhelmed if the risk of NCDs among refugees continues to increase. Thus, it is critical to take primary preventive actions to reduce risk factors for NCDs before they even occur and to take appropriate measures for NCD control at an early stage.

Mental health issues remain a major public health challenge [12]. The WHO reports that “people living with mental health conditions are more likely to face other physical health problems”, such as NCDs [12]. In fact, for refugees, mental disorders become the high burden [12, 13]. Many of them suffer from trauma, depression, anxiety, and post-traumatic stress disorder, caused by their experience of the escape and evacuation from their homeland and fear of persecution [13, 14]. Even after arriving in host countries, refugees continue to be exposed to stressful situations [15–17]. Therefore, there is an urgent need to take some measures to improve and control mental health among refugees.

Tobacco use, physical inactivity, the harmful use of alcohol, and unhealthy diets are the four major modifiable behavioural risk factors for NCDs [18]. According to the WHO, physical inactivity has a wide range of negative impacts on such as the health system, economic development, and quality of life [12]. On the other hand, regular physical activity (PA) not only helps prevent and manage NCDs but also helps prevent diseases and improve mental health and quality of life [13, 18]. PA and physical inactivity affect both mental and physical health [19–21], and regular PA is essential for everyone; refugees are no exception. To collect, analyze, and disseminate data on PA/physical inactivity, many studies have been conducted worldwide using the WHO STEPwise Approach to Chronic Disease Risk Factor Surveillance (STEPS) or the International Physical Activity Questionnaire (IPAQ). However, only a few studies have focused on PA of refugees living in neighbouring countries.

A few previous studies have reported that refugees have a high prevalence of physical inactivity: 50% among Palestine refugees diagnosed with diabetes mellitus living in Jordan [9] and 43% among the Sahrawi people living in refugee camps in Algeria [22].

Updating the prevalence of physical inactivity is significant, particularly for Syrians, because the country’s situation has changed dramatically. Literature review has found that only two studies had reported PA in Syrian refugees living in countries neighbouring Syria: a health status survey of Syrian refugees conducted in Turkey in 2015 [23] and the WHO STEPS survey conducted in Lebanon in 2017 [24]. The STEPS survey conducted in Jordan in 2007 targeted only Jordanians, and refugees were not included. The STEP survey in 2019 was the first one to include Syrian refugees living in Jordan [25]. The results revealed for the first time that the prevalence of physical inactivity among Syrian refugees in Jordan was 21.2%. The survey also showed that 25.7% of Jordanians were physically inactive [25].

Although previous reports have identified various facilitators of and barriers to PA, only a few studies about PA have been conducted among refugees in Arab countries. The present study aimed to determine the prevalence of PA and to identify the facilitators of and barriers to PA among Syrian refugees in Jordan. The findings will enable health policy developers to identify the root causes of some individual and structural risks factors for NCDs and to support strategic planning for multisectoral intervention for better health outcomes for both the host and refugee communities.

Our hypothesis is that the prevalence of physical inactivity among Syrian refugees living in an urban setting is high. We undertook the study in 2017, ahead of the Jordan National STEPS survey for NCD Risk Factors 2019, to determine the prevalence of physical inactivity in Syrian refugees and to assist in solving possible future problems related to NCDs. The 2019 STEPS survey did not include facilitators of and barriers to PA but noted that barriers to PA must be assessed and identified [25]. Therefore, additional studies are valuable for clarifying the facilitators of and barriers to PA, especially among refugees, to make the most of the findings in policy making and project creation.

## Methods

### Study design and settings

**Fig. 1:**
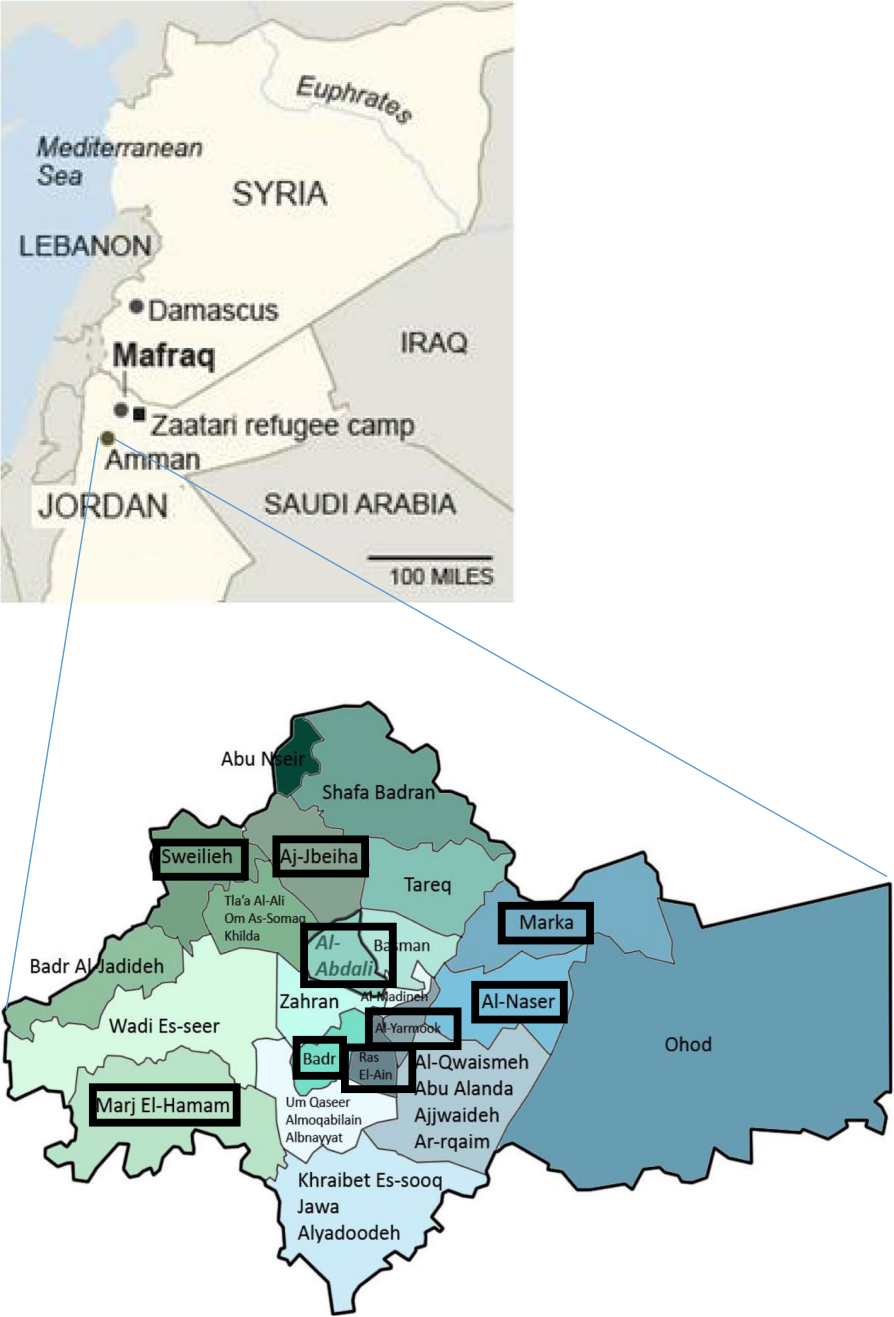
Location of the study site. Above: Location of Jordan and Amman; below: Districts of Amman Governorate. Sources: (above) http://www.nytimes.com/2013/10/06/world/middleeast/as-syrian-refugees-develop-roots-jordan-grows-wary.html. (Below) https://fluswikien.hfwu.de/index.php?title=File:Amman_Districts.jpg

We employed a community-based cross-sectional study design that used two structured questionnaires: the International Physical Activity Questionnaire (IPAQ-short version) [26, 27] and a structured questionnaire. The IPAQ questionnaire comprises questions regarding PA, whereas the structured questionnaire comprises questions regarding sociodemographic characteristics, physical and mental condition, and perception of various aspects of PA. The participants were Syrian refugees living in the following nine districts in the Amman Governorate: Marka, Al-Nasr, Al-Yarmouk, Ras Al-Ein, Bader, Al-Abdali, Sweileh, Jubeiha, and Marj Al-Hamam (Fig. 1). Amman spans over 19 hills, and all the districts in the study area have steep slopes. According to the UNHCR Jordanian staff and Syrian refugee research assistants, Jordanians living in these districts are classified as middle-class. Most of the Syrian refugees in Amman live on the upper floors of the apartments, which have no elevators.

Sample size estimates were based on an expected prevalence of physical inactivity of 10.9%, which was obtained from the mean prevalence values reported in previous studies: physical inactivity of 5.2% among adults in Jordan [28], and physical inactivity among Syrian females (12.3%) and Syrian males (21.1%) [29]. We applied the sample size formula for a prevalence study and a cross-sectional design [30], with a 95% confidence interval (CI) of 1.96 and margin of error of 0.05 for precision. Accordingly, a sample size of 150 was determined. An additional 20% was added to the sample size to overcome attrition due to possible non-response or dropout, resulting in a final sample size of 180 people.

### Participants

Syrian refugees who met the following inclusion criteria were recruited to the study: age 18–64 years (the WHO definition of adult) [18]; living in Amman city; and either registered with the United Nations High Commissioner for Refugees (UNHCR), waiting for their UNHCR registration, or holding a service card issued by the Jordanian Ministry of Interior. Participants were excluded if they had any illness restricting them from performing PA or needed help from others for PA. Those who were hospitalized, pregnant, or lactating were also excluded.

### Data collection and recruitment

Home visits were conducted among Syrian refugees living in Amman from April 13 to May 15, 2017. We used the snowballing method for recruitment: First, our research assistants located a potential candidate for our study. After the participant completed their interview, they were asked to introduce another potential candidate. If the participant failed to introduce another candidate, we asked the neighbours of the participant if any Syrian refugees were living nearby. This process was repeated throughout the whole recruitment period. Participation in the interview was entirely voluntary, and the participants were advised that the study had no risks or benefits and that they could withdraw from participation at any time without penalty. One of our research assistants translated a structured questionnaire from English to Arabic and then pre-tested it among 10 Syrian refugees living in Amman, after which it was used as a self-administered questionnaire. On the other hand, our research assistants administered the IPAQ face-to-face.

Written informed consent was obtained from each participant and/or their legal guardian before administering the questionnaires.

### Ethical approval

This study was approved by the Jordan Ministry of Health, the Health Sector Research and Assessment Committee of UNHCR Jordan, and the Ethical Committee of the School of Tropical Medicine and Global Health, Nagasaki University, Japan (Reference No. 16). All methods were carried out in accordance with relevant guidelines and regulations or Declaration of Helsinki [31].

### Variables and definitions

We collected the following data from the structured questionnaire: 1) sociodemographic characteristics (age, sex, education, occupation, family size, confirmation of registration as a refugee, place of previous residence in Syria), 2) physical condition (health condition, perceived level of PA, diagnosis of hypertension/obesity, and smoking history), 3) mental condition (satisfaction with life in Jordan, perceived stress level), 4) perceived change in the amount of PA, and 5) perceived facilitators and barriers to PA. Age and family size were continuous variables, and the others were categorical variables. The structured questionnaire was based on a previous study that developed lists containing 16 possible facilitators and 25 potential obstacles to PA [32].

In addition to the structured questionnaire, we used the official Arabic short version of the IPAQ [26], which has been examined and validated in previous studies and found acceptable [33, 34]. The IPAQ was used to gather two continuous variables: days and times of each PA (vigorous activity, moderate activity, walking, and sitting) that occurred in the last 7 days. Vigorous PA is defined as an activity that requires intense physical effort and leads to breathing much harder than normal. Heavy lifting and fast bicycling [27] are examples of vigorous activity. Moderate activity is defined as an activity that requires moderate physical effort and leads to breathing somewhat harder than normal [27]. The physical activity score was calculated according to the guidelines of the IPAQ [35].

Metabolic equivalents (METs) for each activity were calculated as continuous variables. The MET is a unit used to estimate of the metabolic cost (i.e., oxygen consumption) of PA. One MET is defined as the energy spent when sitting quietly. MET-minutes per week were calculated according to the intensity of the activity, using the following formulas.

$$\mathrm{Vigorous}\ \mathrm{activity}=\left(8.0\ \mathrm{METs}\ast \mathrm{vigorous}\ \mathrm{activity}\ \mathrm{minutes}\ast \mathrm{vigorous}\ \mathrm{activity}\ \mathrm{days}\right)$$

$$\mathrm{Moderate}\ \mathrm{activity}=\left(4.0\ \mathrm{METs}\ast \mathrm{moderate}\ \mathrm{activity}\ \mathrm{minutes}\ast \mathrm{moderate}\ \mathrm{activity}\ \mathrm{days}\right)$$

$$\mathrm{Walking}\ \mathrm{activity}=\left(3.3\ \mathrm{METs}\ast \mathrm{walking}\ \mathrm{activity}\ \mathrm{minutes}\ast \mathrm{walking}\ \mathrm{activity}\ \mathrm{days}\right)$$

The sum of the METs for all activities (walking, moderate, and vigorous) was also calculated. Each participant’s PA level (high, moderate, or low) was then determined as categorical data according to the IPAQ scoring protocol as follows [36].

Category 1 (Low) is the lowest level of physical activity; those individuals who do not meet the criteria for categories 2 or 3 are considered low/inactive. Any one of the following three criteria is categorized as Moderate (Category2);

- 3 or more days of vigorous activity of at least 20 minutes per day OR.
- 5 or more days of moderate-intensity activity or walking for at least 30 minutes per day OR.
- 5 or more days of any combination of walking, moderate-intensity or vigorous intensity activities achieving a minimum of at least 600 MET-min/week.

Those who meets following two criteria is categorized as Category 3 (High);

- Vigorous-intensity activity on at least 3 days and accumulating at least 1500 MET-minutes/week OR.
- 7 or more days of any combination of walking, moderate-intensity or vigorous intensity activities achieving a minimum of at least 3000 MET-minutes/week.

In analysis of the results of the lists [32] containing multiple facilitators for and barriers to PA, data are presented according to the cut-offs in the WHO global recommendations on PA levels sufficient for health [37]. The recommendations define a healthy level when engaging in at least one of the following: at least 150 minutes of moderate-intensity PA per week or 75 minutes of vigorous-intensity PA, or an equivalent combination of moderate- and vigorous-intensity activity.

### Statistical analysis

The Shapiro–Wilk test was used to assess normality. Mann–Whitney-U test for non-parametric data was used to analyse continuous variables such as the MET score for which the data were not normally distributed. Chi-squared and Cochran–Armitage tests were used to compare each categorical variable with PA level. A backward stepwise logistic regression analysis was conducted to establish independent associations of the significant variables with PA level. Statistical significance was performed at a two-tailed *p-*value of < 0.05. All data were analysed by STATA version 14.

## Results

### Characteristics of the participants

A total of 208 Syrian refugees were interviewed. The following were excluded from the study: lactating women (*n* = 4), individuals without UNHCR registration or a service card from the Jordan Ministry of Interior (*n* = 3), and age greater than 64 years (*n* = 1). Therefore, 200 participants were eligible for the analysis. A further 27 participants did not meet the IPAQ guidelines and were excluded from the analysis of PA [35]. Among the 27 who were excluded, 15 chose the responses “don’t know” or “refuse”, which was regarded as inappropriate for the analysis; and 12 engaged in a total of > 16 hours per day for all activities combined (walking, moderate activity, and vigorous activity time), which was also regarded as inappropriate for the analysis.

The results of the analysis of the characteristics of the participants are summarized in Table 1. Almost half (48.5%) of the participants were male. Mean age was 36.5 years (SD 11.0; range, 18–64 years. There was no significant difference between the sexes in terms of mean age (males, 37.2 ± 11.9 years; females, 35.7 ± 10.1 years). Eighty-four per cent of the study participants were married, the average family size was 5.3 people (SD 2.0), and 2.9% lived alone. The educational levels of males and females was similar, and 44.5% of the participants did not complete primary school education. The majority of males (77.4%) and 5% of females had worked in Syria. After coming to Jordan, 43.3% of males and 6% of females worked outside the home, and 88% of women were housewives. Approximately 50% of males did not work in Jordan, though they had worked as professionals or done other work in Syria.

**Table 1:** General demographic characteristics of the study participants

	No (%) or Means (SD)		
	Total	Male	Female
**Gender**			
**Male**	97 (48.5)		
**Female**	103 (51.05)		
**Age, means (SD), years**	36.5 (11.0)	37.2 (11.9)	35.7 (10.1)
**18–29**	59 (29.5)	30 (30.9)	29 (28.1)
**30–39**	62 (31.0)	25 (25.8)	37 (35.9)
**40–49**	51 (25.5)	26 (26.8)	25 (24.3)
**50–64**	28 (14.0)	16 (16.5)	12 (11.7)
**Marital status**			
**Single**	25 (12.5)	19 (19.6)	6 (5.8)
**Married**	168 (84)	78 (80.4)	90 (87.4)
**Widowed/separated**	7 (3.5)	0 (0.0)	7 (6.8)
**Family size, mean (SD)**	5.3 (2.0)		
**Educational level**			
**Not completed primary**	89 (44.5)	46 (47.4)	43 (41.8)
**Primary school completed**	54 (27.0)	26 (26.8)	28 (27.2)
**Secondary school completed**	37 (18.5)	17 (17.5)	20 (19.4)
**College/University**	20 (10.0)	8 (8.2)	12 (11.7)
**Occupation in Syria**			
**Student**	38 (19.0)	20 (20.6)	18 (17.5)
**Homemaker**	79 (39.5)	0 (0.0)	79 (76.7)
**Professional**	24 (12.0)	22 (22.7)	2 (1.9)
**Employee**	16 (8.0)	15 (15.5)	1 (1.0)
**Agriculture**	3 (1.5)	3 (3.1)	0 (0.0)
**Other**	37 (18.5)	35 (36.1)	2 (1.9)
**Not working**	2 (1.0)	2 (2.1)	0 (0.0)
**Prefer not to answer**	1 (0.5)	0 (0.0)	1 (1.0)
**Occupation in Jordan**			
**Student**	11 (5.5)	5 (5.2)	6 (5.8)
**Homemaker**	91 (45.5)	0 (0.0)	91 (88.3)
**Professional**	9 (4.5)	9 (9.3)	0 (0.0)
**Employee**	17 (8.5)	17 (17.5)	0 (0.0)
**Agriculture**	0 (0.0)	0 (0.0)	0 (0.0)
**Other**	22 (11.0)	16 (16.5)	6 (5.8)
**Not working**	48 (24.0)	48 (49.5)	0 (0.0)
**Prefer not to answer**	2 (1.0)	2 (2.1)	0 (0.0)

Before moving to Jordan, 44.0% of the refugees had lived in Damascus, 32% had lived in Homs, and the remainder were from other governorates. Almost all the participants had fled from Syria because they had felt that their life was in danger. Most participants had lived in Jordan for more than 4 years (79.5%) (Table 2).

**Table 2:** Characteristics of Syrian refugees living in Amman

	Total No (%)	Male No (%)	Female No (%)
The city lived before moving to Amman			
Homs	64 (32.0)	32 (33.0)	32 (31.1)
Aleppo	7 (3.5)	2 (2.1)	5 (4.9)
Damascus	88 (44.0)	41 (42.3)	47 (45.6)
Hamah	10 (5.0)	6 (6.2)	4 (3.9)
Daraa	16 (8.0)	7 (7.2)	9 (8.7)
Idlib	4 (2.0)	2 (2.1)	2 (1.9)
Other	11 (5.5)	7 (7.2)	4 (3.9)
Reason to come to Jordan			
Feel danger	191 (95.5)	91 (93.8)	100 (97.1)
Medical condition	2 (1.0)	2 (2.1)	0 (0.0)
Relatives in Jordan	1 (0.5)	0 (0.0)	1 (1.0)
Other	6 (3.0)	4 (4.2)	2 (2.0)
Duration in Jordan			
0–6 months	1 (0.5)	0 (0.0)	1 (0.5)
1–2 years	1 (0.5)	0 (0.0)	1 (0.5)
2–3 years	9 (4.5)	3 (3.1)	6 (5.8)
3–4 years	30 (15.0)	11 (11.3)	19 (18.4)
More than 4 years	159 (79.5)	83 (85.6)	76 (73.8)

The self-reported physical and mental conditions of the participants were listed in Table 3. More than half of the study participants (53.5%) felt able to control the condition of their health. Most had a high or moderate perceived level of PA, more than a third (33.5%) of all participants had been diagnosed with hypertension, and 15.0% with obesity. More than half of males (68.0%) were current smokers (cigarettes, shisha, or both), and 90% of females had never smoked. Approximately 40% of all participants felt stressed after coming to Jordan, and 54.5% were satisfied with the current situation in Jordan. Almost half of all participants perceived a decrease in PA living in Jordan compared with that in Syria.

**Table 3:** Physical and mental conditions of Syrian refugees living in Amman

	Total No (%)	Male No (%)	Female No (%)
**Perceived health condition**			
**Under control**	107 (53.5)	51 (52.6)	56 (54.4)
**Not under control**	91 (45.5)	45 (46.4)	46 (44.7)
**Prefer not to answer**	2 (1.0)	1 (1.0)	1 (1.0)
**Perceived level of physical activity**			
**High**	46 (23.0)	25 (25.8)	21 (20.4)
**Moderate**	117 (58.5)	48 (49.5)	69 (67.0)
**Low**	37 (18.5)	24 (24.7)	13 (12.6)
**Hypertension**			
**Yes**	67 (33.5)	31 (32.0)	36 (35.0)
**No**	130 (65.0)	64 (66.0)	66 (64.1)
**Prefer not to answer**	3 (1.5)	2 (2.1)	1 (1.0)
**Obesity**			
**Yes**	30 (15.0)	8 (8.2)	22 (21.4)
**No**	168 (84)	89 (91.8)	79 (76.7)
**Prefer not to answer**	2 (1.0)	0 (0.0)	2 (1.9)
**Smoking**			
**Current smoker**	77 (38.5)	66 (68.0)	11 (10.7)
**Never smoker**	123 (61.5)	31 (32.0)	92 (89.3)
**Satisfaction with current situation in Jordan**			
**Yes**	109 (54.5)	45 (46.4)	64 (62.1)
**No**	67 (33.5)	41 (42.3)	26 (25.2)
**Prefer not to answer**	24 (12.0)	11 (11.3)	13 (12.6)
**Feel stress after coming to Joran**			
**Yes**	77 (38.5)	43 (44.3)	34 (33.0)
**Neutral**	38 (19.0)	14 (14.4)	24 (23.3)
**No**	71 (35.5)	32 (33.0)	39 (37.9)
**Prefer not to answer**	14 (7.0)	8 (8.2)	6 (5.8)
**The perceived change in amount of physical activity during living in Jordan compared with lived in Syria**			
**Increased**	69 (34.5)	30 (30.9)	39 (37.8)
**Decreased**	106 (53.0)	57 (58.8)	49 (47.5)
**No change**	17 (8.5)	7 (7.2)	10 (9.7)
**Not sure**	8 (4.0)	3 (3.1)	5 (4.8)

### Patterns of physical activity

**Fig. 2:**
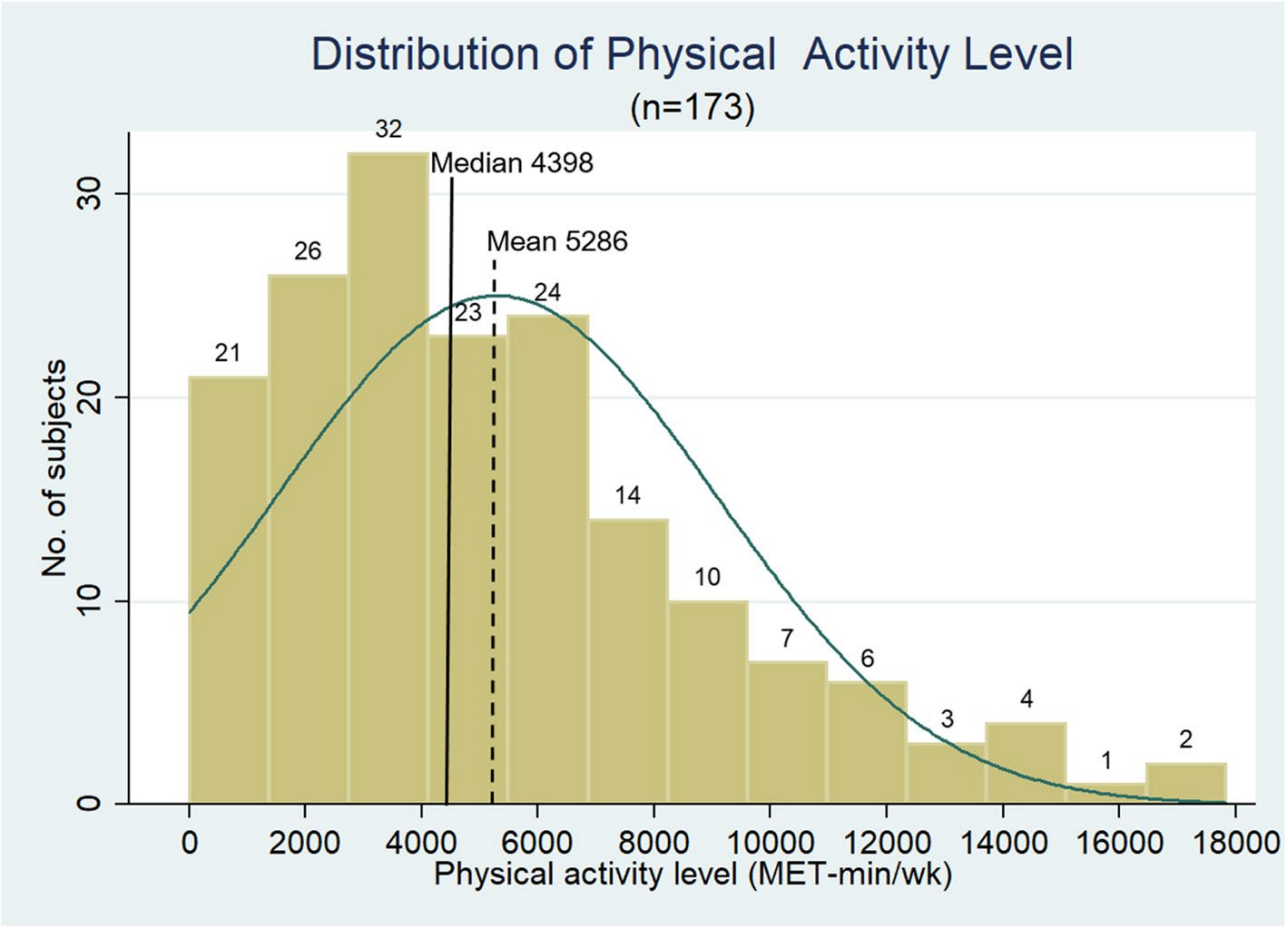
Distribution of PA level. The IPAQ was used to measure PA levels. MET was calculated according to the method described in the Data management section and is shown as MET-min/wk

The total PA score for all participants (*n* = 173) was non-parametric (right-skewed) in distribution (Fig. 2). The median MET was 4398 min/wk. (range, 0–17,838; IQR, 0, 7386).

According to the IPAQ scoring system [36], PA level was identified as follows: high at 65.9%, moderate at 26.0% and low at 8.1%. There was no statistically significant difference in terms of sex (*p* = 0.68) (Table 4).

**Table 4:** IPAQ score among Syrian refugees

Physical Activity Level	Total No (%) *n* = 173	Male No (%) *n* = 79	Female No (%) *n* = 94	*p*-value^a^
High	114 (65.9)	52 (65.8)	62 (66.0)	
Moderate	45 (26.0)	22 (27.8)	23 (24.5)	
Low	14 (8.1)	5 (6.3)	9 (9.6)	*p* = 0.68

^a^ Chi-square test

There was no significant difference in total MET score between males and females (*p* = 0.78), but activity-specific scores showed a significant difference by sex. Median MET scores for vigorous activity and moderate-intensity activity were both higher in females (*p* < 0.01, *p* < 0.05), whereas the median MET scores for walking activity and sedentary time were both higher in males (*p* < 0.01) (Table 5).

**Table 5:** Patterns of physical activity levels among Syrian refugees

	Participants No (%)	Median (IQR)	Range	*p*-value^a^
All activity (MET-min/wk)				
Total	173	4398 (0, 7386)	0–17,838	
Male	79 (45.7)	4066.5 (0, 7572)	0–15,813	
Female	94 (54.3)	4673.5 (0, 6657)	0–17,838	*p* = 0.78
Vigorous activity (MET-min/wk)				
Total	182	960 (0, 2880)	0–10,080	
Male	85 (46.7)	480 (0, 2400)	0–10,080	
Female	97 (53.3)	1920 (0, 2880)	0–10,080	*p* < 0.01
Moderate activity (MET-min/wk)				
Total	185	1440 (0, 1440)	0–5760	
Male	86 (46.5)	660 (0, 2520)	0–5760	
Female	99 (53.5)	1440 (0, 3360)	0–5040	*p* < 0.05
Walking activity (MET-min/wk)				
Total	186	693 (0, 1386)	0–4158	
Male	87 (46.8)	1039.5 (0, 2772)	0–4158	
Female	99 (53.2)	396 (0, 1188)	0–4158	*p* < 0.01
Sedentary time (minutes)				
Total	181	180 (0, 300)	0–960	
Male	83 (45.9)	240 (60, 600)	60–960	
Female	98 (54.1)	180 (0, 240)	0–960	*p* < 0.01

*IQR* Interquartile range (first-third quartiles)

*MET-min/wk* Metabolic equivalent-minutes/week

^a^ Mann-Whitney U test

### Factors associated with physical inactivity

The results of the analysis of factors associated with PA lists in Table 6. Among the age groups, the prevalence of physical inactivity was highest in participants aged 18 to 30 years (11.8%), lowest in those aged 45–64 years (4.4%) (*p* = 0.21; crude OR = 0.35; 95%CI: 0.07–1.82) and was 7.8% in those aged 31–44 years (*p* = 0.45; crude OR = 0.63; 95%CI: 0.19–2.09).

**Table 6:** Bivariate analysis and logistic regression analysis of predictors of physical inactivity according to physical activity level

	Physically Inactive No (%) *n* = 14	Physically active No (%) *n* = 159	Crude OR (95%CI)	Adjusted OR (95%CI)	*p*-value^b^
Gender					
Male	5 (6.3)	74 (93.7)	Ref		
Female	9 (9.6)	85 (90.4)	1.57 (0.50–4.88)		*p* = 0.44
Age					
18–30	6 (11.8)	45 (88.2)	Ref		
31–44	6 (7.8)	71 (92.2)	0.63 (0.19–2.09)		*p* = 0.45
45–64	2 (4.4)	43 (95.6)	0.35 (0.07–1.82)		*p* = 0.21
Occupation in Jordan					
Not working	5 (10.2)	44 (89.8)	Ref		
Homemaker	7 (8.4)	76 (91.6)	0.81 (0.24–2.71)		*p* = 0.73
Working	2 (4.9)	39 (95.1)	0.45 (0.08–2.46)		*p* = 0.35
Educational level					
< Primary school	11 (8.9)	113 (91.1)	Ref		
> Secondary school	3 (6.1)	36 (93.9)	0.67 (0.18–2.51)		*p* = 0.55
Marital status					
Single	2 (11.1)	16 (88.9)	Ref		
Married/Widow	12 (7.7)	143 (92.3)	0.67 (0.14–3.28)		*p* = 0.62
The place before coming to Amman^a^					
West	3 (5.5)	52 (94.5)	Ref		
North	1 (5.9)	16 (94.1)	1.08 (0.11–11.15)		*p* = 0.95
South	10 (9.9)	91 (90.1)	1.90 (0.50–7.23)		*p* = 0.34
Duration in Jordan					
< 4 years	6 (16.2)	31 (83.8)	Ref		
4 years or longer	8 (5.9)	128 (94.1)	3.10 (1.00–9.58)		*p* < 0.05
Perceived health condition					
Not controlled	6 (7.5)	74 (92.5)	Ref		
Controlled	8 (8.6)	85 (91.4)	1.16 (0.38–3.50)		*p* = 0.87
Perceived level of physical activity					
Active	10 (7.0)	132 (93.0)	Ref		
Inactive	4 (12.9)	27 (87.1)	1.96 (0.57–6.69)		*p* = 0.28
Hypertension					
No	9 (7.9)	105 (92.1)	Ref		
Yes	5 (8.5)	54 (91.5)	1.08 (0.34–3.38)		*p* = 0.85
Obesity					
No	10 (6.9)	136 (93.1)	Ref		
Yes	4 (14.8)	23 (85.2)	2.37 (0.08–8.18)		*p* = 0.16
Smoking					
Never smoker	12 (10.7)	100 (89.3)	Ref		
Current smoker	2 (3.3)	59 (96.7)	0.28 (0.06–1.31)		*p* = 0.28
Satisfaction with the current situation in Jordan					
Yes	8 (8.3)	89 (91.7)	Ref		
No	3 (5.4)	53 (94.6)	1.96 (0.47–8.16)		*p* = 0.35
Prefer not to answer	3 (15.0)	17 (85.0)	0.63 (0.16–2.48)		*p* = 0.51
Feel stress after coming to Joran					
No	5 (8.1)	57 (91.9)	Ref		
Yes	9 (8.1)	102 (91.9)	1.01 (0.32–3.15)		*p* = 0.99
The perceived change in the amount of physical activity in Jordan compared to Syria					
Increased	2 (3.3)	58 (96.7)	Ref	Ref	
Decreased	7 (7.7)	84 (92.3)	2.40 (0.48–12.05)	3.00 (1.27–7.26)	
No change/Not sure	5 (22.7)	17 (77.3)	8.53 (1.52–47.9)		*p* < 0.05

Data presented as no. (%) unless otherwise indicated

*OR* Odds ratio, *CI* Confidence interval, *Ref* Reference group

^a^ West: Homs. North: Aleppo, Hamah, Idlib. South: Damascus, Darra, Other

^b^ Cochran-Armitage, Chi-square test

The proportion of physical inactivity was significantly higher in those who had stayed in Jordan for less than 4 years (16.2%) than in those who had stayed in Jordan for 4 years or longer (5.9%) (*p* = 0.05; crude OR = 3.10; 95%CI: 1.00–9.58).

Stepwise logistic regression analysis revealed only perceived change in the amount of PA as an independent factor associated with physical inactivity (*p* < 0.05, adjusted OR = 3.00; 95%CI: 1.27–7.26).

### Perceived facilitators of and barriers to physical activity

The result of the analysis of the perceived of and barriers to PA are shown in Table 7. Among all participants, the most frequent facilitators of PA were (in descending order) “psychological wellbeing”(49.7%), “prevent diseases”(46.8%), “relieve tension”(43.4%), “self-dependence”(42.2%), and “promote and maintain health”(41.6%). A larger number of physically active participants indicated that “weight control/obesity prevention”(41.5%) and “psychological wellbeing”(52.8%) were facilitators of PA (*p* < 0.01). In contrast, the proportion of those who indicated “no benefits” was significantly higher among physically inactive participants (28.6%) compared with those who were physically active (10.7%) (*p* < 0.05).

**Table 7:** Perceived facilitators of physical activity among Syrian refugees

Facilitator	Total No (%) *n* = 173	Physically Active No (%) *n* = 159	Physically Inactive No (%) *n* = 14	*p*-value ^a^	Meeting WHO recsNo (%) *n* = 96	Not meeting WHO recs No (%) *n* = 77	*p*-value ^a^
Promote and maintain health	72 (41.6)	68 (42.8)	4 (28.6)	0.30	38 (39.6)	34 (44.2)	0.54
Improve body image and shape	54 (31.2)	52 (32.7)	2 (14.3)	0.15	33 (34.4)	21 (27.3)	0.32
Improve muscle power	17 (9.8)	17 (10.7)	0 (0.0)	0.20	11 (11.5)	6 (7.8)	0.42
Spent free times	42 (24.3)	40 (25.2)	2 (14.3)	0.36	27 (28.1)	15 (19.5)	0.19
Weight control/obesity prevention	67 (38.7)	66 (41.5)	1 (7.1)	0.01	45 (46.9)	22 (28.6)	0.01
Psychological wellbeing	86 (49.7)	84 (52.8)	2 (14.3)	0.01	49 (51.0)	37 (48.1)	0.70
Recreation	29 (16.8)	28 (17.6)	1 (7.1)	0.32	19 (19.8)	10 (13.0)	0.23
Prevent diseases	81 (46.8)	76 (47.8)	5 (35.7)	0.39	48 (50.0)	33 (42.9)	0.35
Improve mentality and intellectuality	35 (20.2)	35 (22.0)	0 (0.0)	0.05	25 (26.0)	10 (13.0)	0.03
Companionship with others	43 (24.9)	43 (27.0)	0 (0.0)	0.03	28 (29.2)	15 (19.5)	0.14
Socializing	37 (21.4)	37 (23.3)	0 (0.0)	0.04	22 (22.9)	15 (19.5)	0.58
Fun and enjoyment	32 (18.5)	31 (19.5)	1 (7.1)	0.25	19 (19.8)	13 (16.9)	0.62
Improve sleeping	37 (21.4)	36 (22.6)	1 (7.1)	0.18	23 (24.0)	14 (18.2)	0.36
Self-dependence	73 (42.2)	71 (44.7)	2 (14.3)	0.03	46 (47.9)	27 (35.1)	0.09
Relieve tension	75 (43.4)	72 (45.3)	3 (21.4)	0.08	45 (46.9)	30 (39.0)	0.30
No benefits	21 (12.1)	17 (10.7)	4 (28.6)	0.05	13 (13.5)	8 (10.4)	0.53
Median (IQR) no. of facilitators		4 (1, 7)	2 (1, 3)		4 (1, 7.5)	3 (1, 5)	

*IQR* Interquartile range (first-third quartiles)

^a^ Chi-square test

Among 77 participants who did not meet the WHO recommendations on PA for health, around 45% chose “psychological wellbeing”(48.1%) and “promote and maintain health”(44.2%) as perceived facilitators. In contrast, more than half of those considered to meet these recommendations chose “psychological wellbeing”(51.0%) and “prevent diseases”(50.0%).

The perceived barriers to PA are listed in Table 8. Among all participants, “high cost”(57.8%) was the most common perceived barrier to PA. In those categorized as physically inactive, “time limitation”(42.9%) was the greatest perceived barrier.

**Table 8:** Perceived barriers to physical activity among Syrian refugees

Barrier	Total No (%) *n* = 173	Physically Active No (%) *n* = 159	Physically Inactive No (%) *n* = 14	*p*-value ^a^	Meeting WHO recs No (%) *n* = 96	Not meeting WHO recs No (%) *n* = 77	*p*-value ^a^
Time limitations	75 (43.4)	69 (60.5)	6 (42.9)	0.97	46 (47.9)	29 (37.7)	0.18
Lack of accessible and suitable sports places	39 (22.5)	36 (31.6)	3 (21.4)	0.92	16 (16.7)	23 (29.9)	0.04
Lack of safe sports places	36 (20.8)	32 (28.1)	4 (28.6)	0.46	19 (19.8)	17 (22.1)	0.71
Lack of support and encouragement from others	42 (24.3)	41 (36.0)	1 (7.1)	0.12	25 (26.0)	17 (22.1)	0.55
Lack of friends to encourage me	31 (17.9)	30 (26.3)	1 (7.1)	0.27	20 (20.8)	11 (14.3)	0.26
Have other important priorities	74 (42.8)	71 (62.3)	3 (21.4)	0.09	43 (44.8)	31 (40.3)	0.55
Lack of sports programs that suit my physical fitness	13 (7.5)	13 (11.4)	0 (0.0)	0.27	9 (9.4)	4 (5.2)	0.30
Not interested in sports	19 (11.0)	16 (14.0)	3 (21.4)	0.19	11 (11.5)	8 (10.4)	0.82
Lack of motivation	12 (6.9)	11 (9.6)	1 (7.1)	0.98	8 (8.3)	4 (5.2)	0.42
High cost	100 (57.8)	95 (83.3)	5 (35.7)	0.08	62 (64.6)	38 (49.4)	0.04
Lack of sports skills	9 (5.2)	9 (7.9)	0 (0.0)	0.36	4 (4.2)	5 (6.5)	0.49
Fear of failure in sports competition	14 (8.1)	13 (11.4)	1 (7.1)	0.89	4 (4.2)	10 (13.0)	0.04
Fear of injury	25 (14.5)	24 (21.1)	1 (7.1)	0.42	13 (13.5)	12 (15.6)	0.70
Fear of deterioration of physical illness	19 (11.0)	17 (14.9)	2 (14.3)	0.68	9 (9.4)	10 (13.0)	0.45
Nobody to care for my family	63 (36.4)	60 (52.6)	3 (21.4)	0.22	42 (43.8)	21 (27.3)	0.03
Feeling tired on physical activity	33 (19.1)	32 (28.1)	1 (7.1)	0.24	17 (17.7)	16 (20.8)	0.61
Ignorance about benefits of sports	4 (2.3)	4 (3.5)	0 (0.0)	0.55	4 (4.2)	0 (0.0)	0.07
Prefer not to attend sports place	5 (2.9)	5 (4.4)	0 (0.0)	0.50	3 (3.1)	2 (2.6)	0.84
Lack of or low physical power	24 (13.9)	22 (19.3)	2 (14.3)	0.96	11 (11.5)	13 (16.9)	0.31
Feeling unable to practice sports adequately	18 (10.4)	17 (14.9)	1 (7.1)	0.68	7 (7.3)	11 (14.3)	0.13
Objection of others	5 (2.9)	5 (4.4)	0 (0.0)	0.50	3 (3.1)	2 (2.6)	0.84
Body cannot tolerate physical activity	30 (17.3)	28 (24.6)	2 (14.3)	0.75	16 (16.7)	14 (18.2)	0.79
Unsuitable (hot or cold) weather	15 (8.7)	14 (12.3)	1 (7.1)	0.83	6 (6.3)	9 (11.7)	0.21
Previous bad experience with physical sport activity	9 (5.2)	9 (7.9)	0 (0.0)	0.36	5 (5.2)	4 (5.2)	1.00
No barriers	11 (6.4)	11 (9.6)	0 (0.0)	0.31	8 (8.3)	3 (3.9)	0.24
Median (IQR) no. of barriers		3 (1, 5)	2.5 (1,3)		3 (1, 5)	3 (1, 5)	

*IQR* Interquartile range (first-third quartiles)

^a^ Chi-square test

There was no significant relationship between level of PA and proportion of each perceived barrier to PA (*p* = 0.08–0.98). Among the physically inactive participants, at least one barrier to PA was reported (median 2.5).

“High cost”(64.6%) (49.4%), “have other important priorities”(44.8%) (40.3%), and “time limitation”(47.9%) (37.7%) were the most commonly reported barriers in both those who met and those who did not meet the WHO recommendations on PA for health.

## Discussion

This study provides valuable insights into the level of PA and the facilitators and barriers to PA among Syrian refugees living in Amman. Regarding the first key results, the study revealed that most Syrian refugees had a high level of PA; only 8.1% were physically inactive. This finding is lower than the result of the STEPS survey conducted in Jordan in 2019: the prevalence of insufficient PA among Syrian respondents was 21.2%, and that of Jordanians was 25.7% [25]. The STEPS survey in Syria in 2003 reported 32.9% of the Syrian did not meet these recommendations [38]. Other previous studies also reported refugees to be physically inactive in their host countries [23, 39]. This insufficiency could be attributed to several reasons: 1) the duration of refuge in Jordan spent by the Syrian. More than 79% of the participants had lived in Jordan for more than 4 years, and only few participants had lived in Jordan for a shorter period. According to a recent study reported that newly arrived refugees consider PA as a lesser priority [40], the PA level may depend on the length of stay in the host country. Therefore, fewer participants might have been classified as physically inactive in this study; 2) the difference in the target areas. The STEPS survey in Jordan in 2019 was a national survey that included Zarqa, Irbid, and Mafraq governorates in addition to Amman, whereas the present survey was only conducted in the Amman governorate; 3) Amman is a hilly city, making physical movement demanding. The WHO and other studies have reported geography as a critical factor influencing PA [41, 42]; Future regular studies including a broader sample and region are necessary to better compare the differences in PA levels across different lengths of stay and areas of residence in Jordan or any other host countries [25]; 4) the living environment of a refugee. A 2014 study that measured PA level across 5 refugee camps for Sahrawi people in Algeria showed that 43% of the participants were physically inactive [22]. Compared to living in a refugee camp, where movement is restricted to within the camp, urban refugees have fewer restrictions on movement. In addition, although the participants in this study live in a city, they are located far from the urban centre or in inconvenient places, which requires them to walk long distances to go shopping or visit administrative organizations such as the UNHCR office. However, no previous studies have compared the PA level between urban refugees and refugees in camp settings. Thus, we believe further studies are necessary to validate this finding; 5) potential motivation for participants to engage in PA [40]. The previous study reported that intrinsic motivation has been positively linked with PA [43]. Nearly half of the participants reported “promote and maintain health”(41.6%), “psychological wellbeing”(49.7%), and “relieve tension”(43.4%) as perceived facilitators, suggesting that spontaneous factors such as walking for a change of mood may have increased their PA level. Furthermore, although the participants in this study were not asked in detail about PA, some of the homes we visited for the survey had equipment for strength training and/or sports. To precisely identify the facilitators of PA, future study determining the facilitators by the level of PA is required.

Our results show that the PA level varied significantly by sex when considered in terms of METs. The levels of vigorous and moderate activity were higher among women than among men. These results are not consistent with those reported in most previous studies, which found that men were more likely to engage in vigorous activity because of social pressure and cultural norms [44]. The occupation status of the refugees can partially explain this finding. More than half of males did not work while almost all the females were housewives. Women, particularly mothers, believed that household chores were a type of exercise that could substitute for practicing sport [45]. The present study showed that men spent more time walking and sitting than did women, which is consistent with the finding reported in the international study by Bauman et al. (2009). That study noted that according to cultural norms, men had more opportunities to walk, either to the mosque to pray or to accompany women when they went out [46].

With regard to the predictors of PA, our analysis revealed that the self-perceived amount of PA was a significant indicator. In addition, those who did not report a change in their PA after arrival in Jordan were more physically inactive than those who did. This is likely associated with the finding reported by Morrison et al. (2017) that a negative mood (manifested here as indifference to PA status and amount of PA) is related to low PA levels [47].

We did not find a significant association between age and obesity and PA in this study. However, previous studies have reported that PA decreases with increasing age of a generation as their physical strength declines with age, making it more difficult for them to achieve normal PA levels [23, 48, 49]. The unique circumstance of the refugees requires the older adults to engage in labour work to support their families. The proportion of physically inactive refugees was higher among those with obesity than among those without. Several studies have reported higher levels of physical inactivity among obese participants [50, 51]. One plausible explanation of our result is the underestimation of the prevalence of obesity in this study at 15.0%, which is lower than the Syrian national representative (26.0%) [52]. This could be attributed to the use of self-report data instead of medical data [53].

The answers to questions about the facilitators of PA focused on physical and mental wellbeing. Almost half of the participants perceived that “psychological wellbeing” and “prevent diseases” could facilitate PA. “Relieve tension”, “self-dependence”, and “promote and maintain health” were mainly perceived as facilitators by physically active participants. Although a previous study supported our finding of “promote and maintain health” as a frequently reported facilitator among Egyptian University students, “relieve tension” was not a popular facilitator among them [32]. The difference between the main facilitators in our study and those among Egyptian students might be because of the refugees experiencing stress after arriving in Amman that makes them choose psychological factors as major facilitators of PA. This is in line with previous studies that found that positive beliefs, negative mood, and belief change had an association with change in PA level [32, 54, 55]. Given that psychological factors can effect a change in PA, they should be fully utilized in promotional programs to guarantee their success. More participants will engage in PA if they associate it with experiencing positive feelings and removing negative ones. Furthermore, our study found an association between a perceived facilitator “psychological wellbeing” and PA level. This result is consistent with that of previous studies that PA also plays an important role in improving the mental health of refugees [20, 21, 56]. Although no significant association between the PA level and the perceived facilitator “relieve tension” that was chosen by 43.4% of all participants was seen, PA plays an important role in improving mental health.

Regarding the barriers to PA, “high cost” and “time limitation” were the most significant barriers, consistent with previous reports [32, 44, 57]. As most refugees are in an economically difficult situation, “high cost” is the most common barrier [58]. “Time limitation”, which is likely the most common reason worldwide, was associated with other barriers such as having other priorities and being the sole source of care for the family [59]. Apart from other previously identified barriers such as a lack of knowledge of facilities in the local area, of the recommended levels of PA and of its health benefits (which could be addressed through awareness campaigns), time management skills, and reduction of PA-associated costs such as promotion of on-line based PA and distribution of coupons for using gyms should be considered by any program promoting PA [40]. Although no significant association between the PA level and the perceived barrier “Have other important priorities” was identified in this study, a similar barrier is reported as “competing priorities” by previous studies on identifying barriers and facilitators of PA among asylum seekers [40]. Future studies on determining the exact time limitations and costs that hinder PA and identifying factors taking higher priority over PA, among refugees in the host country, are required to devise strategies to promote PA among refugees according to its facilitators and barriers.

In this survey, we asked the participants to choose facilitators and barriers from a limited set of choices. However, other facilitators and barriers exist. In particular, regarding barriers, previous research reported that differences in language, culture, and religious beliefs could also be barriers [59–61]. Guerin et al. (2003) reported that even when refugees have the opportunity to participate in PA programs in the host country, lack of understanding of the local language and poor literacy make following instruction challenging for them [39]. Experiences of discrimination and stigmatization may also affect PA [16, 62]. Gele A. et al. (2015) found that Somali women in Norway felt shame and stigma when they wanted to access health facilities such as the gym and swimming pool. Therefore, conducting additional research among urban refugees in the host country exploring more barriers and facilitators such as the aforementioned ones will aid in better understanding. Qualitative research is also essential to identifying potential facilitators and barriers. For the participants of this study and the refugees in Amman city, “high cost” and “time limitation” could be solved by setting up a place to provide opportunities for refugees to engage in PA in or near institutions such as the UNHCR where refugees visit for procedures. Offering different PA programs depending on the level of PA with free smartphone applications could be a practical solution [63].

This study had several limitations. First, the participants did not represent the entire population of interest as they were selected using a snowballing method, which might have introduced selection bias. Thus, our results cannot be generalized. Second, the results obtained could be underestimated or overestimated because some questions, such as amount of PA and diagnosis of hypertension and obesity, were answered through self-report measures. In addition, the question in IPAQ about PA in the last 7 days may have introduced recall bias. Furthermore, cross-sectional studies have the inherent disadvantage of being unable of establishing cause and effect. Moreover, the participants were not enquired about the details of PA, family size, cost, and the use of facilities that require fees, such as gyms. Such detailed information may have been useful in exploring the association with the PA level. With regard to family size, having family members requiring care, such as infants or elderly people, could have influenced the activity levels. Finally, we did not consider the effect size. Despite these limitations, our study adds to the body of literature on refugees’ PA and its distinctive facilitators and barriers, which can aid future studies that plan to promote PA among refugee populations.

## Conclusions

In this study, we determined the PA level among Syrian refugees living in Amman, Jordan. This study also identified the most common facilitators of PA were “psychological wellbeing” and “prevent diseases”, and the most common barriers were “time limitation” and “high cost”. Our findings provide a valuable baseline for future studies to examine PA level and to verify its possible facilitators and barriers among refugees. Flexible PA programs promoted according to the PA level of refugees through free smartphone applications could improve their PA level and mental health, which would contribute to the prevention of NCDs among them. Furthermore, a mainstreaming of refugees into national interventions that aims to improve PA could apply for local people as well. Urgent action to developing strategies to support both physical and mental health among refugees by promoting PA is critical. Refugees should be surveyed more frequently, given their vulnerable and the changing environment.

## Supplementary Information

**Additional file 1.** Questionnaire.

## Data Availability

The datasets used and/or analysed during the current study are available from the corresponding author on reasonable request due to privacy.

## Abbreviations

GPAQ: Global Physical Activity Questionnaire
IPAQ: International Physical Activity Questionnaire
METs: Metabolic equivalents
MET-min/wk.: Metabolic equivalents-minutes per week
NCDs: Non-communicable Diseases
PA: Physical activity
STEPS: WHO STEPwise Approach to Chronic Disease Risk Factor Surveillance
UNHCR: United Nations High Commissioner for Refugees
UNRWA: United Nations Relief and Works Agency for Palestine Refugees in the Near East
WHO: World Health Organization
