# Supplementary material for: Assessment of physical activity and its facilitators and barriers among Syrian refugees living in Amman City, Jordan: a cross-sectional study
Source: BMC Public Health. 2022 Sep 12;22:1732. doi: 10.1186/s12889-022-14064-1 (PMC9469621; doi:10.1186/s12889-022-14064-1)
Supplement: Supplementary file 1 — Additional file 1. Questionnaire. [file 12889_2022_14064_MOESM1_ESM.pdf]

Appendix3: Questionnaire

Questionnaire

		code
**Location**	1.Amman city	
**No of list**		
**Date**	/ / 2017	
**Name of research assistance**		

1.Basic Demographics Characteristics

No	Question	Response	Code
**1-1**	What sex are you?	1. Male<br>2. Female	
**1-2**	How old are you?	___________ years old	
**1-3**	What is your occupation now?	1. Student<br>2. Housewife<br>3. Craftman<br>4. employee<br>5. Agriculture<br>6. Other<br>7. Retired<br>8. Not working<br>0. Prefer not to answer	
**1-4**	What is the highest level of education you have completed?	1. No formal schooling<br>2. Less than primary school<br>3. Primary school completed<br>4. Secondary school completed<br>~~5. High school completed~~<br>5. College/University completed<br>6. Postgraduate degree<br>0. Prefer not to answer	
**1-5**	What is your marital status?	1. Single<br>2. Married<br>3. divorced	

		4. No longer married	
**1-6**	How many in your family? (include yourself)	___________ member(s)	
**1-7**	Are you living with others who needs care from others now?	1. Yes<br>2. No<br>0. Prefer not to answer	

2. Characteristics as a refugee in Jordan

No	Question	Response	code
**2-1**	Where in Syria are you from?	1. Homs<br>2. Aleppo<br>3. Damascus<br>4. Hamah<br>5. Dara'a<br>6. Idlib<br>7. Latakiya<br>8. Other<br>0. Prefer not to answer	
**2-2**	What was your occupation in Syria?	1. Student<br>2. Housewife<br>3. Professional/semi professional<br>4. employee<br>5. Agriculture<br>6. Other<br>7. Retired<br>8. Not working<br>0. Prefer not to answer	
**2-3**	What is a reason you come to Jordan?	1. Because you feel the danger<br>2. Because of your medical condition<br>3. Because there are relatives in Jordan<br>4. Other<br>0. Prefer not to answer	
**2-4**	How long have you been in Jordan?	1. 0-6 months<br>2. 6-12 months	

		3. more than 1 year and less than 2 years<br>4. more than 2 years and less than 3 years<br>5. more than 3 years and less than 4 years<br>6. more than 4 years<br>0. Prefer not to answer	
**2-5**	Are you currently registered with UNHCR?	1. Yes, I am registered with UNHCR<br>2. NO, I am not registered with UNHCR<br>3. I am waiting for completion of register<br>0. Prefer not to answer	
**2-6**	Do you obtain a service card by Jordan ministry of Interior?	1. Yes, I have a service card<br>2. No, I do not have a service card<br>0. Prefer not to answer	

3. Physical condition

**No**	**Question**	**Response**	**code**
**3-1**	Would you consider your health condition "under control" right now?	1. Yes<br>2. No<br>0. Prefer not to answer	
**3-2**	How do you think about your level of physical activity?	1. High<br>2. Moderate<br>3. Low	
**3-3**	Have you diagnosed as hypertension?	1. Yes<br>2. No<br>0. Prefer not to answer	
**3-4**	Have you diagnosed as obesity?	1. Yes<br>2. No<br>0. Prefer not to answer	
**3-5**	Do you currently smoke any tobacco products, such as algeere, cigarettes, cigars or pipes?	1. Yes<br>2. No<br>0. Prefer not to answer	

4. Mental condition

No	Question	Response	code
**4-1**	Are you satisfied with current situation in Jordan?	1. Yes<br>2. No<br>0. Prefer not to answer	
**4-2**	Do you feel stress after coming to Jordan?	1. Yes<br>2. Neutral<br>3. No<br>0. Prefer not to answer	

5. Facilitators and Barriers to physical activity

No	Question	Response	code
**5-1**	Which are benefits for you to do physical activity?<br><br>*could be multiple answer	1. Promote and maintain health<br>2. Improve body image and shape<br>3. Improve muscle power<br>4. Spent free times<br>5. Weight control/obesity prevention<br>6. Psychological wellbeing<br>7. Recreation<br>8. Prevent diseases<br>9. Improve mentality and intellectuality<br>10. Companionship with others<br>11. Socializing<br>12. Fun and enjoyment<br>13. Improve sleeping<br>14. Self-dependence<br>15. Relieve tension<br>16. No benefits	
**5-2**	Which is the most barrier for you to do physical activity?<br><br>*could be multiple answer	1. Time limitations<br>2. Lack of accessible and suitable sports places<br>3. Lack of safe sports places<br>4. Lack of support and encouragement from others<br>5. Lack of friends to encourage me<br>6. Have other important priorities	

		7. Lack of sports programs that suit my physical fitness<br>8. Not interested in sports<br>9. Lack of motivation<br>10. High cost<br>11. Lack of sports skills<br>12. Fear of failure in sports competition<br>13. Fear of injury<br>14. Fear of deterioration of physical illness<br>15. Nobody to care for my family<br>16. Feeling tired on physical activity<br>17. Ignorance about benefits of sports<br>18. Prefer not to attend sports place<br>19. Lack of or low physical power<br>20. Feeling unable to practice sports adequately<br>21. Objection of others<br>22. Body cannot tolerate physical activity<br>23. Unsuitable (hot or cold) weather<br>24. Previous bad experience with physical sport activity<br>25. No barriers	
5-3	Would you consider the amount of your physical activity in Jordan increase/decrease, comparing with that of Syria?	1. the amount of physical activity increased in Jordan than in Syria<br>2. the amount of physical activity decreased in Jordan than in Syria<br>3. the amount of physical activity not change<br>4. Not sure	
